# Supplementary material for: Mapping the evolution of fertility support policies in China: A content and instrumental analysis
Source: PLoS One. 2025 Oct 9;20(10):e0332137. doi: 10.1371/journal.pone.0332137 (PMC12510515; doi:10.1371/journal.pone.0332137)
Supplement: S1 Appendix — (ZIP) [file pone.0332137.s001.zip › S1 Appendix. 226 original policy documents/32-国家卫生健康委办公厅关于做好托育机构复托相关工作的通知(FBM-CLI-4-344286).doc]

国家卫生健康委办公厅关于做好托育机构复托相关工作的通知

**发布部门：** [国家卫生健康委员会](https://www.pkulaw.com/law/chl?Aggs.IssueDepartment=60321) [机构沿革](javascript:void(0))

**发文字号：**国卫办人口函〔2020〕469号

**发布日期：**2020.06.15

**实施日期：**2020.06.15

**时效性：** [现行有效](https://www.pkulaw.com/law/chl?Aggs.TimelinessDic=01)

**效力级别：** [部门工作文件](https://www.pkulaw.com/law/chl?Aggs.EffectivenessDic=XE0303)

**法规类别：** [机关工作综合规定](https://www.pkulaw.com/law/chl?Aggs.Category=00301)

国家卫生健康委办公厅关于做好托育机构复托相关工作的通知
（国卫办人口函〔2020〕469号）

各省、自治区、直辖市及新疆生产建设兵团卫生健康委：
　　为全面贯彻党中央关于抓紧抓实抓细常态化疫情防控、做好当前经济社会发展各项工作的决策部署，安全有序推进托育机构恢复入托，现就有关事项通知如下：
　　一、坚持属地管理的原则，按照当地党委政府的统一部署，认真评估中小学开学复课情况，参照幼儿园复园时间，确定托育机构复托时间。

　　二、认真组织检查评估托育机构复托前各项准备工作，未达到《托幼机构新冠肺炎疫情防控技术方案（修订版）》（国卫办疾控函〔2020〕363号）各项要求的，不得复托。

　　三、加强托育机构日常健康管理，严格实行工作人员和婴幼儿健康状况“日报告”、“零报告”制度，落实入托婴幼儿晨（午）检、全日观察、因病缺勤病因追查及登记等防控措施，做好场所通风换气、清洁灭菌等工作，切实保障婴幼儿生命安全和身体健康。

　　四、主动协调编制、民政、市场监管等部门，加快推进托育机构登记和备案工作。县级卫生健康行政部门应在2020年7月31日前，完成托育机构备案信息系统管理用户注册。

　　五、加强调查研究，及时了解托育机构面临的现实困难和突出问题，积极协调发展改革、财政、人力资源社会保障、金融、税务等部门，在落实好国家各项优惠政策的同时，结合本地实际，研究制订促进托育机构发展的支持政策。

国家卫生健康委办公厅
2020年6月15日

© 北大法宝：（[www.pkulaw.com](https://www.pkulaw.com/)) 专业提供法律信息、法学知识和法律软件领域各类解决方案。北大法宝为您提供丰富的参考资料，正式引用法规条文时请与标准文本核对。
欢迎查看所有[产品和服务](https://www.pkulaw.com/helps/2.html)。法宝快讯：[如何快速找到您需要的检索结果？](https://www.pkulaw.com/helps/70.html)[法宝](https://www.pkulaw.com/helps/69.html)V6有何新特色？

扫描二维码阅读原文

**原文链接：**<https://www.pkulaw.com/chl/e13fa70c536668f8bdfb.html>
